# Squamous cell carcinoma on an arteriovenous fistula scar: case report

**DOI:** 10.1590/1677-5449.202200622

**Published:** 2023-06-30

**Authors:** Vinicius Tadeu Ramos da Silva Grillo, Pedro Luciano Mellucci, Marina Moraes Lopes Soares, Nathalia Dias Sertorio, Rodrigo Gibin Jaldin, Marcone Lima Sobreira, Eloisa Bueno Pires de Campos, Matheus Bertanha

**Affiliations:** 1 Universidade Estadual Paulista Júlio de Mesquita Filho - UNESP, Botucatu, SP, Brasil.

**Keywords:** arteriovenous fistula, squamous cell carcinoma, skin neoplasms

## Abstract

The main type of access used for hemodialysis is the arteriovenous fistula (AVF) because it offers superior patency and lower complication rates when compared to other hemodialysis accesses. We report the case of a 69-year-old female patient with chronic kidney disease on dialysis secondary to hypertensive nephrosclerosis with a radiocephalic AVF in the left upper limb created 9 years previously. Two years previously, she had undergone a kidney transplant and was taking immunosuppressants. A crusted lesion developed on her left forearm with onset 3 months before presentation and she underwent an excisional biopsy that revealed a well-differentiated and superficially invasive squamous cell carcinoma, with lateral and deep surgical margins free from neoplasia. At 1-year follow-up, the patient showed no signs of neoplastic recurrence.

## INTRODUCTION

Worldwide, approximately 3 million people are treated with hemodialysis, or around 70% of patients with end-stage chronic kidney disease (CKD). Although hemodialysis can greatly increase patient survival, it is associated with considerable morbidity and mortality and is extremely expensive for health care systems.^1^

The main type of access used for hemodialysis is the arteriovenous fistula (AVF) because it offers superior patency and lower complication rates when compared to other accesses for hemodialysis. Use of an AVF is associated with longer survival, lower rates of infection and hospital admissions, and lower costs.^2^

Patients eligible for creation of an AVF include patients with end-stage CKD not yet on dialysis, with severe and progressive kidney damage or with glomerular filtration rates of 15 to 20 mL/min/1.73m^2^. Patients in transition from other types of renal substitution such as peritoneal dialysis, with failed grafts, or who are already on hemodialysis using venous catheters should be considered for an AVF.^3^

The most common complications associated with AVFs are thrombosis (15-25%), stenosis (14-42%), congestive heart failure (12.2-17%), ischemic neuropathy (1-10%), steal syndrome (2-8%), aneurysms (5-6%), and infections (2-3%).^4,5^ Squamous cell carcinoma is not generally one of the entities listed in literature reviews. However, the correlation between scarring of tissue that repeatedly suffers traumas and spindle cell degeneration does exist and is known as a Marjolin ulcer (MU).^6^ This entity can occur in relation to AVF, since its hyperproliferative nature is associated with sites that undergo multiple punctures.

This study was duly evaluated and approved by the Research Ethics Committee (Ethics Appraisal Submission Certificate 57892922.2.0000.5411, decision number 5.381.959).

## CASE REPORT

A white, female, 69-year-old patient presented at the dermatology clinic at the Hospital das Clínicas, Faculdade de Medicina de Botucatu, Universidade Estadual Paulista (FMB-UNESP), because of a crusted lesion on her left forearm with onset 3 months previously. She had systemic arterial hypertension and dialytic CKD, with etiology secondary to hypertensive nephrosclerosis, and had undergone creation of a radio-cephalic AVF in her left arm 9 years previously. Two years previously she had received a successful kidney transplant and her AVF had been deactivated 6 months previously because kidney function was normal.

She was on immunosuppressant therapy because of her postoperative kidney transplant status, taking tacrolimus 0.1 mg/kg/day, sirolimus 2 mg, and prednisone 5 mg/day.

**Figure 1:**
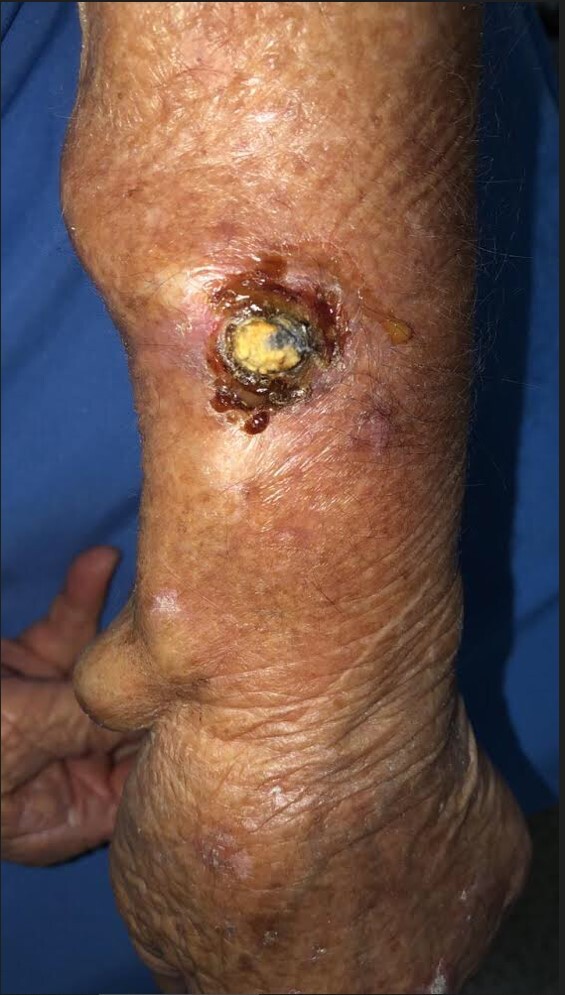
Lesion with an infiltrating base and crusted surface measuring 2 x 2 cm on the left forearm, located on the puncture site used for cannulation of the dilated cephalic vein.

Physical examination found a lesion with an infiltrating base and crusted surface measuring 2 x 2 cm on the left forearm, located at the puncture site used for cannulation of the dilated cephalic vein (Figure 1). Arterial physical examination found that the ulnar artery pulse was present and the radial artery pulse was absent. Despite dilation of the cephalic vein, no thrill was detected on palpation or auscultation, suggesting that the surgical AVF ligation procedure had been successful.

The patient underwent an ambulatory excisional biopsy procedure and the specimen was sent for anatomopathological analysis. She was prescribed antibiotic therapy during the postoperative period, with oral cephalexin 500 mg every 6 hours and daily dressings with topical mupirocin for 7 days. The sutures were removed after 14 days when she was prescribed topical collagenase because of discrete dehiscence of the operating wound, after which the operating wound healed fully.

The anatomopathological analysis revealed that the lesion was a well-differentiated and superficially invasive squamous cell carcinoma (SCC) with lateral and deep surgical margins free from neoplasia.

At a 1-year follow-up assessment, the patient’s operating wound had healed completely, cephalic vein dilatation had receded, and she was free from signs of relapse of the neoplastic lesion.

## DISCUSSION

The first description of chronic ulcerous lesions was published in 1828, by the French surgeon Jean-Nicholas Marjolin, who described them as arising in burns. In 1903, Da Costa correlated them with malignant transformation. Nowadays, a MU refers to a malignant tumor, generally an SCC, which develops over a chronic wound.^7,8^

Although the majority of cases of MU occur over burns, they can also occur over several other types of lesions: pressure ulcers, venous ulcers, irradiated tissues, diabetic ulcers, osteomyelitis, burns, vaccination scars, hidradenitis suppurativa, pilonidal cysts, and discoid lupus. Transformation of different types of wounds into malignant neoplasm is multifactorial.^8^

The physiopathogenesis that provokes malignant transformation can be explained by a process of repetitive traumas and immunological suppression caused by the low level of vascularization of scar tissue, which creates an area of unstable tissue, since the chronic irritation is associated with cell proliferation and release of pro-mitotic toxins.^9,10^

This tissue is poorly vascularized, giving the cells generated by neoplastic transformation of the wound resistance to apoptosis induced by the immune system, particularly in immunodepressed patients, who are at increased risk. The patient in question was taking immunosuppressant medication after a kidney transplant (tacrolimus, sirolimus, and prednisone).

In the present case, squamous cell carcinoma was identified, which is the most common pathology in MUs, accounting for 75 to 90% of cases. There are other histological types that may possibly be associated, such as basal cell carcinoma, melanoma, sarcoma, and basalioma.^8^ Carcinomas that emerge in scarred areas are associated with worse prognosis because of aggression and local infiltration, which should be confirmed with histological examination after excisional biopsy.

The first choice treatment for MU is surgery with careful excision of the lesion to avoid tumor dissemination, including a minimum safety margin of 1 cm. In specific cases in which there is regional lymphadenopathy, regional lymph node clearance by lymphadenectomy is indicated, and, in extreme cases with joint or bone invasion, amputation may be needed. If surgical resection is impossible, radiotherapy may be indicated, alone or in combination with chemotherapy.^8^

## CONCLUSIONS

Professionals working at hemodialysis services should always remember the possibility of a diagnosis of SCC associated with repetitive trauma due to use of an AVF. The association with repeated trauma is well described, but the relationship with dialysis patients is discussed little, considering the particularly common trauma linked to puncture of fistulas.

Training of professionals to identify suspect lesions should go beyond vascular surgeons and nephrologist physicians and should be extended to the technicians and nurses who are generally responsible for punctures, with the objective not only of identifying at-risk lesions, but also to remind them of the importance of performing punctures along the entire length of the fistula.
