# Supplementary material for: Squamous cell carcinoma on an arteriovenous fistula scar: case report
Source: J Vasc Bras. 2023 Jun 30;22:e20220062. doi: 10.1590/1677-5449.202200622 (PMC10421574; doi:10.1590/1677-5449.202200622)
Supplement: Translation

# Translation: Carcinoma espinocelular sobre cicatriz de fístula arteriovenosa: relato de caso

**DOI:** 10.1590/1677-5449.202200621

**Authors:** Vinicius Tadeu Ramos da Silva Grillo, Pedro Luciano Mellucci, Marina Moraes Lopes Soares, Nathalia Dias Sertorio, Rodrigo Gibin Jaldin, Marcone Lima Sobreira, Eloisa Bueno Pires de Campos, Matheus Bertanha

**Affiliations:** 1 Universidade Estadual Paulista Júlio de Mesquita Filho - UNESP, Botucatu, SP, Brasil.

**Keywords:** fístula arteriovenosa, carcinoma de células escamosas, neoplasias cutâneas

## Abstract

A fístula arteriovenosa (FAV) é o principal acesso para hemodiálise devido à sua patência superior e menores índices de complicação quando comparada aos demais acessos para hemodiálise. Relatamos o caso de uma paciente do sexo feminino, de 69 anos, com doença renal crônica dialítica secundária a nefroesclerose hipertensiva com FAV radio-cefálica no membro superior esquerdo realizada há 9 anos. Há 2 anos, foi submetida a transplante renal e fazia uso de imunossupressores. Evoluiu com aparecimento de lesão crostosa em antebraço esquerdo há 3 meses, foi submetida a biópsia excisional, e foi evidenciado carcinoma espinocelular bem diferenciado e superficialmente invasivo, com margens cirúrgicas laterais e profundas livres de neoplasia. No seguimento de 1 ano, a paciente não apresentava sinais de recidiva neoplásica.

## INTRODUÇÃO

No mundo, aproximadamente 3 milhões de pessoas, ou cerca de 70% dos pacientes com doença renal crônica (DRC) terminal, são tratadas com hemodiálise. Embora capaz de proporcionar um aumento expressivo de sobrevida ao paciente, a morbimortalidade associada à hemodiálise é alta, além de ser um método extremamente dispendioso para os sistemas de saúde^1^ .

A fístula arteriovenosa (FAV) é o principal acesso para a hemodiálise devido à sua perviedade superior e aos menores índices de complicação quando comparada aos demais acessos. Está relacionada a maior sobrevida, menores índices de infecção e de hospitalização e custos reduzidos^2^ .

Entre os pacientes elegíveis para a confecção da FAV, encontram-se pacientes com DRC terminal em regimes pré-dialítico, com lesões renais graves e progressivas ou com taxas de filtração glomerular entre 15 e 20 mL/min/1,73m^2^. Pacientes em transição de outros regimes de substituição renal como diálises peritoneais, falha de enxertos ou que já se encontram em regime de hemodiálise com cateteres venosos devem ser considerados^3^ .

As principais complicações associadas à FAV são tromboses (15-25%), estenoses (14-42%), insuficiência cardíaca congestiva (12,2-17%), neuropatias isquêmicas (1-10%), síndrome do roubo (2-8%), aneurismas (5-6%) e infecções (2-3%)^4,5^ . Observa-se que o carcinoma espinocelular não é uma entidade comumente descrita nas revisões literárias. A correlação entre a cicatrização tecidual recorrentemente abalada por traumatismos de repetição e degenerações espinocelulares existe e é denominada úlcera de Marjolin (UM)^6^ . Essa entidade pode estar presente na FAV, visto que a sua natureza hiperproliferativa é associada ao local que recebe múltiplas punções.

Este estudo foi devidamente avaliado e aprovado pelo Comitê de Ética em Pesquisa (Certificado de Apresentação de Apreciação Ética 57892922.2.0000.5411, parecer número 5.381.959).

## RELATO DO CASO

Uma paciente do sexo feminino, branca, de 69 anos de idade, deu entrada no ambulatório de dermatologia do Hospital das Clínicas da Faculdade de Medicina de Botucatu da Universidade Estadual Paulista (FMB-UNESP) devido a lesão crostosa em antebraço esquerdo com início há 3 meses. Ela era portadora de hipertensão arterial sistêmica, com histórico de DRC dialítica com etiologia secundária a nefroesclerose hipertensiva; havia sido submetida à confecção de FAV radio-cefálica no membro superior esquerdo há 9 anos. Há 2 anos, foi submetida a transplante renal com sucesso, e, devido à função renal normal, foi feita a desativação da FAV há 6 meses.

Apresentava-se em uso de terapia imunossupressora devido ao pós-operatório de transplante renal, com uso de tacrolimus 0,1 mg/kd/dia, sirolimus 2 mg e prednisona 5 mg/dia.

Ao exame físico, apresentava lesão de base infiltrativa e superfície crostosa medindo 2 x 2 cm no antebraço esquerdo, localizada no local de punção para canulação da veia cefálica dilatada (Figura 1). Ao exame físico arterial, foram observados pulso presente na artéria ulnar e pulso ausente na artéria radial. Apesar da dilatação da veia cefálica, não se identificava frêmito à palpação ou ausculta, sugerindo efetividade do procedimento de ligadura cirúrgica da FAV.

**Figura 1:** Lesão de base infiltrativa e superfície crostosa medindo 2 x 2 cm no antebraço esquerdo, localizada no local de punção para canulação da veia cefálica dilatada.

A paciente foi submetida a procedimento ambulatorial de biópsia excisional da lesão, e o material foi encaminhado para análise anatomopatológica. No pós-operatório, foi prescrita antibioticoterapia com cefalexina 500 mg por via oral a cada 6 horas e curativo diário com mupirocina tópica durante 7 dias. Os pontos de sutura foram retirados em 14 dias, e, devido a discreta deiscência de ferida operatória, optou-se pela prescrição de colagenase tópica, evoluindo com cicatrização total da ferida operatória.

A análise anatomopatológica constatou tratar-se de um carcinoma espinocelular (CEC) bem diferenciado e superficialmente invasivo, com margens cirúrgicas laterais e profunda livres de neoplasia.

Em reavaliação após 1 ano, a paciente apresentava-se com cicatrização total da ferida operatória, com regressão da dilatação da veia cefálica e sem sinais de recidiva da lesão neoplásica.

## DISCUSSÃO

A descrição de uma lesão ulcerosa crônica foi realizada pela primeira vez em 1828, pelo cirurgião francês Jean-Nicholas Marjolin em queimados, e correlacionada à transformação maligna em 1903 por Da Costa. Atualmente, a UM refere-se a um tumor maligno, geralmente o CEC, que se diferencia sobre uma ferida crônica^7,8^ .

Embora ocorra na maioria das vezes sobre queimaduras, a UM pode ocorrer em diversos tipos de lesões: úlceras de pressão, úlceras venosas, tecidos irradiados, úlceras diabéticas, osteomielites, queimaduras, cicatrizes de vacinação, hidradenite supurativa, cistos pilonidais e lúpus discoide. A transformação de diferentes tipos de feridas em neoplasia maligna é multifatorial^8^ .

A fisiopatogenia que leva à transformação maligna pode ser explicada a partir de traumas repetitivos e supressão imunológica em decorrência da baixa vascularização do tecido cicatricial, formando uma área com tecido instável, uma vez que a irritação crônica se associa à proliferação celular e à liberação de toxinas pró-mitóticas^9,10^ .

Devido à vascularização pobre do tecido, as células decorrentes da transformação neoplásica da ferida apresentam resistência à apoptose induzida pelo sistema imune, sobretudo em pacientes imunossuprimidos, que apresentam risco aumentado. A paciente em questão encontrava-se em uso de medicações imunossupressoras após transplante renal (tacrolimus, sirolimus e prednisona).

No presente relato, foi identificado o CEC, que é a patologia mais frequente nas UMs, ocorrendo em 75 a 90% dos casos. Existem outros possíveis tipos histológicos associados, como carcinoma basocelular, melanoma, sarcoma e basalioma^8^ . Carcinomas que surgem em áreas de cicatrizes estão associados a um pior prognóstico devido à agressividade e à infiltração local, devendo ser confirmados com exame histológicos após biópsia excisional.

A primeira opção de tratamento na UM é a cirurgia com excisão da lesão com cautela para evitar disseminação tumoral, devendo incluir margem de segurança mínima de 1 cm. Em casos específicos em que há linfadenopatia regional, indica-se complementação com esvaziamento ganglionar regional por meio de linfadenectomia, e, em casos extremos, com invasão articular ou óssea, a amputação pode ser indicada. Na impossibilidade de ressecção cirúrgica, pode ser indicada radioterapia exclusiva ou combinada à quimioterapia^8^ .

## Conclusão

O diagnóstico de CEC associado ao trauma de repetição para utilização de FAV deve ser sempre lembrado pelos profissionais atuantes nos serviços de hemodiálise. A associação entre trauma de repetição é bem descrita; entretanto, sua relação com pacientes em diálise, considerando o trauma especialmente comum na punção de fístulas, é pouco discutida.

O treinamento profissional para identificação de lesões suspeitas deve extrapolar médicos-cirurgiões vasculares e nefrologistas, devendo ser estendido para técnicos e enfermeiros, comumente responsáveis pelas punções, com o objetivo não apenas de identificar lesões de risco, mas também de buscar memorar a importância de punções em toda a extensão da fístula.
